# Exogenous SA Affects Rice Seed Germination under Salt Stress by Regulating Na^+^/K^+^ Balance and Endogenous GAs and ABA Homeostasis

**DOI:** 10.3390/ijms23063293

**Published:** 2022-03-18

**Authors:** Zhiguo Liu, Chunyang Ma, Lei Hou, Xiuzhe Wu, Dan Wang, Li Zhang, Peng Liu

**Affiliations:** 1 College of Plant Protection, Shandong Agricultural University, Tai’an 271000, China; 17863800846@163.com (Z.L.); hl1695779182@163.com (L.H.); wuxzdee@163.com (X.W.); a736615291@163.com (D.W.); lilizhang324@163.com (L.Z.); 2 State Key Laboratory of Crop Biology, Shandong Agricultural University, Tai’an 271000, China; machunyang100@163.com

**Keywords:** salicylic acid, salt stress, rice, germination, ion balance, hormone homeostasis

## Abstract

Salinity reduces agricultural productivity majorly by inhibiting seed germination. Exogenous salicylic acid (SA) can prevent the harm caused to rice by salinity, but the mechanisms by which it promotes rice seed germination under salt stress are unclear. In this study, the inhibition of germination in salt-sensitive Nipponbare under salt stress was greater than that in salt-tolerant Huaidao 5. Treatment with exogenous SA significantly improved germination of Nipponbare, but had little effect on Huaidao 5. The effects of exogenous SA on ion balance, metabolism of reactive oxygen species (ROS), hormone homeostasis, starch hydrolysis, and other physiological processes involved in seed germination of rice under salt stress were investigated. Under salt stress, Na^+^ content and the Na^+^/K^+^ ratio in rice seeds increased sharply. Seeds were subjected to ion pressure, which led to massive accumulation of H_2_O_2_, O_2_^−^, and malonaldehyde (MDA); imbalanced endogenous hormone homeostasis; decreased gibberellic acid (GA1 and GA4) content; increased abscisic acid (ABA) content; inhibition of α-amylase (EC 3.2.1.1) activity; and slowed starch hydrolysis rate, all which eventually led to the inhibition of the germination of rice seeds. Exogenous SA could effectively enhance the expression of *OsHKT1;1*, *OsHKT1;5*, *OsHKT2;1* and *OsSOS1* to reduce the absorption of Na+ by seeds; reduce the Na^+^/K^+^ ratio; improve the activities of SOD, POD, and CAT; reduce the accumulation of H_2_O_2_, O_2_^−^, and MDA; enhance the expression of the GA biosynthetic genes *OsGA20ox1* and *OsGA3ox2*; inhibit the expression of the ABA biosynthetic gene *OsNCED5*; increase GA1 and GA4 content; reduce ABA content; improve α-amylase activity, and increase the content of soluble sugars. In summary, exogenous SA can alleviate ion toxicity by reducing Na^+^ content, thereby helping to maintain ROS and hormone homeostasis, promote starch hydrolysis, and provide sufficient energy for seed germination, all of which ultimately improves rice seed germination under salt stress. This study presents a feasible means for improving the germination of direct-seeded rice in saline soil.

## 1. Introduction

Abiotic stress factors, such as drought, salinity, extreme temperature, and heavy metals, can severely affect plant growth and reduce plant productivity by more than 50% [1]. Salt stress is a major abiotic stress factor that is caused by the high concentration of sodium (Na^+^) and chloride ions (Cl^−^) in soil [2]. Soils are described as saline soils when their electrical conductivity reaches 4 dS/m (equivalent to 40 mM NaCl), which can inhibit plant growth significantly [3]. Statistically, more than 800 million hectares of land (about 6% of the total land area) worldwide are affected by salt [3]. Economic losses in agriculture due to salt stress are estimated to be up to USD 27 billion a year [4]. It is estimated that by 2050, >50% of the arable land will be affected by salt stress globally [5]. Most crops are glycophytic plants; i.e., they are very sensitive to salt stress, and thus, when NaCl concentrations reach 100 to 200 mM, their growth is completely inhibited, or the crop may even die [6]. This poses a significant obstacle to meeting the goal of increasing agricultural output by 70% by the year 2050 [7,8].

Rice (*Oryza sativa* L.) is one of the main grain crops; it feeds more than half of the world’s population [9]. It is estimated that ~30% of the land used for rice cultivation is affected by salinization worldwide [10]. As rice is sensitive to salt stress, such land significantly reduces crop yield and quality [11,12]. Seed germination is not only the beginning of plant growth and development, but is also a key link that determines the quality of seedling growth [13]. However, seed germination is susceptible to many factors, such as drought [14], light [15], temperature [16], saline–alkaline [17], and heavy metals [18]. Unsuitable environmental conditions can readily compromise seed germination rate, leading to weak seedling growth. On the one hand, direct damage caused to seed germination by salt stress includes osmotic stress, which restricts water absorption and accumulation by seed, and the production of reactive oxygen species (ROS) is induced, resulting in oxidative damage. On the other hand, salt stress leads to the accumulation of Na^+^ and Cl^−^ in seeds. Excess sodium in plant cells is a toxic effect that leads to phytohormone imbalance, generation of (ROS), and changes in membrane permeability [19]. Many studies have shown that salt stress may significantly inhibit the germination of rice seeds [20,21]. However, in recent years, many Asian countries have grown rice by direct seeding rather than transplanting to save time and money [22]. Therefore, it has become crucial to devise means of promoting the germination of rice seeds under salt stress to ensure sufficient rice yield in the future.

It is well known that gibberellic acid (GA) is the most important plant hormone to break dormancy and promote seed germination [23]. Liu et al. [24] demonstrated that a reduction in α-amylase activity by lowering the content of bioactive GA is one of the pathways by which NaCl inhibits germination of rice seeds. This inhibitory effect of salts can be alleviated by the application of exogenous GA3. Nakaune et al. [25] found that GA played an even stronger role than abscisic acid (ABA) in regulating the germination of tomato seeds under short-term salt stress. At present, more than 130 types of GAs are known in plants, fungi, and bacteria. However, most GAs in plants are biologically inactive and exist either as a precursor of a biologically active form or as an inactivated metabolite. Only GA1, GA3, GA4, and GA7 are the major bioactive GAs in plants [26]. GA3 is bioactive in most higher plants, but in rice, GA1 and GA4 are the main bioactive GAs [27]. Homeostasis of bioactive GAs in plants is regulated by biosynthesis and inactivation pathways [28]. Furthermore, genes encoding GA synthase include *CPS*, *KS*, *KO*, *KAO*, *GA20ox*, and *GA3ox* [29], where *GA2ox* regulates plant growth by inactivating endogenous bioactive GA [30].

In addition to GA, ABA also plays an important role in the regulation of seed germination by promoting seed dormancy [31]. Chen et al. [32] demonstrated that salts inhibit seed germination by decreasing endogenous GA content and increasing endogenous ABA content. Similar to GA homeostasis, ABA homeostasis is regulated by ABA biosynthesis and catabolism genes [33]. For ABA biosynthesis in higher plants, 9-cis epoxycarotenoid dioxygenase (NCED) is a key enzyme [34], while ABA 8ʹ-hydroxylases encoded by *CYP707A* gene catalyzes ABA decomposition [35]. The ABA content in wheat seeds of the *aba8′oh1a* and *aba8′oh1d* double mutants was significantly higher than that in the wild type during seed germination, which enhanced seed dormancy [36]. However, the expression of *TsNCED1* in seeds of germination-insensitive cultivars before the harvest was higher than germination-sensitive cultivars before harvest [37]. Li et al. [38] found that under salt stress, exogenous melatonin reduces the expression of the ABA biosynthetic genes *NCED1* and *NCED3*, and increases the expression of the ABA catabolic genes *LbCYP707A1* and *LbCYP707A2*, thus reducing ABA accumulation to alleviate the inhibitory effect of salt on seed germination of *Limonium bicolor*. Blue-light-induced secondary dormancy in barley seeds has been shown to increase the expression of *HvNCED1* and *HvNCED2* and decrease the expression of *HvABA8’OH-1*, leading to increased ABA accumulation [39].

The energy required for seed germination and early growth of seedlings is mainly provided by the hydrolysis of starch stored in the endosperm [40]. α-Amylase (EC 3.2.1.1) is a key catalytic enzyme in starch hydrolysis [41]. Kaneko et al. [42] proved that GA may induce the expression of RAmy1A, which promotes the synthesis of α-amylase, thus accelerating starch hydrolysis. The activity of α-amylase is lowered by salt stress, but is restored by treatment with exogenous GA3 to some extent [24]. Therefore, decreased α-amylase activity induced by salt stress may be caused by two pathways. On the one hand, it may be due to the decrease in bioactive GA content; on the other hand, the accumulation of Na^+^ may cause ion toxicity, deactivating α-amylase.

A large uptake of Na^+^ changes membrane polarization and alters the electron transport chain, which elevates the rates of ROS generation [43]. Accumulation of large amounts of ROS causes oxidative damage to the cell by attacking macromolecules, such as DNA, RNA, lipids, and proteins, which compromises membrane integrity and, finally, leads to oxidative stress and retardation of plant growth and development [44]. Previous studies have shown that ROS accumulation in seeds under salt stress inhibits germination [45,46]. Superoxide dismutase (SOD), peroxidase (POD), and catalase (CAT) are the key enzymes involved in the metabolism of ROS. An increase in their activities contributes to the removal of ROS [47].

Salicylic acid (SA) is a small phenolic substance that is widely present in plants. It is also an important plant hormone that is involved in various developmental stages and physiological mechanisms in plants [48]. Xie et al. [49] found that under normal conditions, SA may reduce the activity of α-amylase by regulating the expression of *HvWRKY38*, thus inhibiting seed germination. However, under salt stress, exogenous SA reduces the loss of water from the cell by increasing the accumulation of osmotic substances, which decreases intracellular water potential [50]. At the same time, treatment of SA decreases the accumulation of Na^+^ and Cl^−^, while increasing K^+^ content, which helps maintain ion homeostasis in plants [51]. This hormone also can increase the activity of antioxidant enzymes and the accumulation of nonenzymatic antioxidants, leading to reduced oxidative damage [52], In addition, it can promote seed germination [53], photosynthesis [54], and nutrient metabolism [55] to improve growth and reproductive development; therefore, SA ultimately improves the yield and quality of crops under salt stress [56]. Research studies have shown that treatment with exogenous SA may promote the germination of rice seeds under salt stress. However, these studies only studied the germination phenotype, while the mechanism of germination was not explored [57,58]. To clarify the mechanisms by which SA promotes rice seed germination under salt stress, in this study, we investigated the mechanism of exogenous SA regulating ion balance, hormone homeostasis, ROS, and starch metabolism during rice seed germination under salt stress, providing a theoretical basis for alleviating plant salt stress with exogenous SA.

## 2. Results

### 2.1. Exogenous SA Promoted the Germination of Rice Seeds under Salt Stress

**Figure 1:**
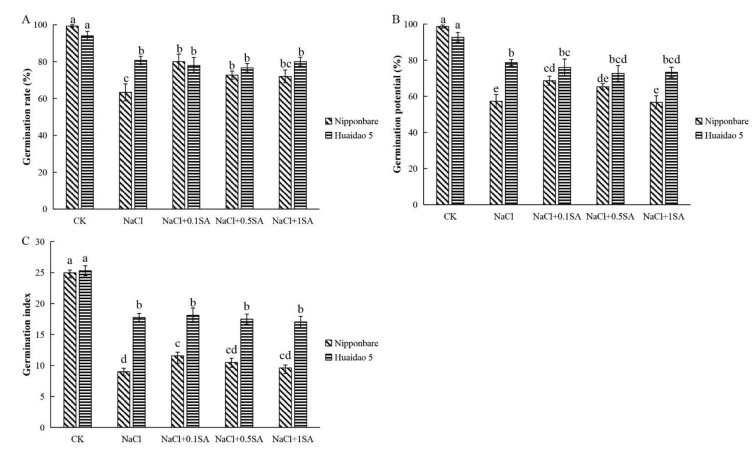
Effects of different concentrations of SA on the germination of rice seeds (Nipponbare and Huaidao 5) under salt stress. Distilled water was used in the CK treatment; the concentration of NaCl was 150 mM (NaCl), and the dosage of salicylic acid (SA) was 0.1, 0.5, or 1 mM (NaCl + SA). The seeds were incubated in a 90 mm culture dish with two layers of filter paper for 5 days, followed by calculation of (**A**) germination rate, (**B**) germination percentage, and (**C**) germination index. Data are presented as means ± SE. Different lowercase letters indicate significant differences between treatments (*p* < 0.05).

**Figure 2:**
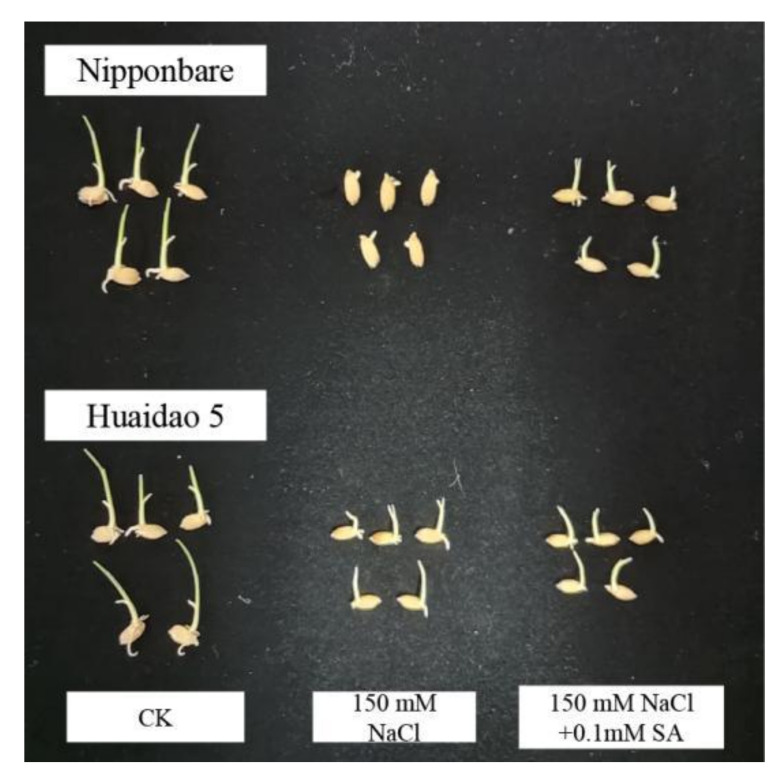
Effects of NaCl and SA on root and shoot growth of Nipponbare and Huaidao 5.

After incubation for 120 h, the germination rate (GR) of both genotypes of rice seeds under CK treatment was close to 100% (Figure 1A). However, under 150 mM NaCl stress, the GR of Huaidao 5 reached 80%, while that of Nipponbare was only 63.33% (Figure 1A). Exogenous SA treatment effectively alleviated the inhibitory effect of salt stress on the germination of rice seeds. Upon treatment with 0.1, 0.5, and 1 mM SA, the germination rate of Nipponbare increased by 26.32%, 14.74%, and 13.68%, respectively. However, different concentrations of SA had no significant effect on the germination rate of Huaidao 5 under salt stress (Figure 1A). Salt stress had a severe inhibitory effect on the germination potential (GP) and germination index (GI) of rice seeds, especially the salt-sensitive Nipponbare genotype. When the seeds of Nipponbare and Huaidao 5 were exposed to 150 mM NaCl, their GP decreased by 41.89% and 15.11%, respectively, as compared to that of CK (Figure 1B). Treatment with 0.1 and 0.5 mM SA significantly increased GP by 19.77% in Nipponbare, but had no significant effect in Huaidao 5. Salt stress also significantly reduced the GI of seeds. Although exogenous SA did not affect the GI of Huaidao 5, 0.1 mM SA increased the GI of Nipponbare by 28.33% (Figure 1C). In addition, 0.1 mM SA also promoted the growth of root and shoot of Nipponbare under salt stress, but did not promote the growth of Huaidao 5 (Figure 2).

### 2.2. Exogenous SA Maintained Ion Homeostasis in Rice Seeds under Salt Stress

**Figure 3:**
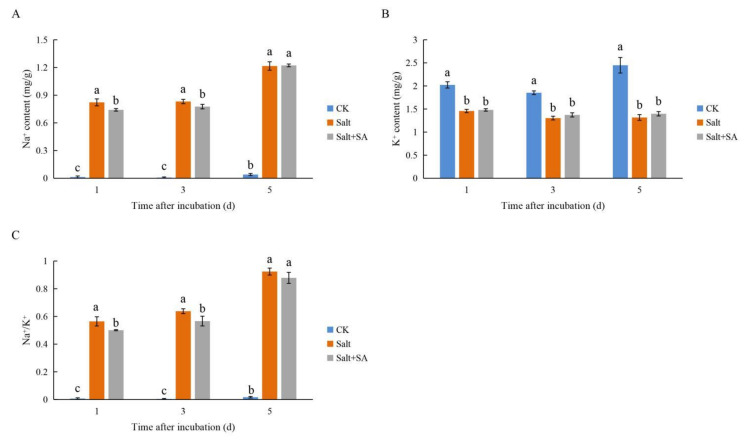
Effects of exogenous salicylic acid (SA) on ion accumulation in rice seeds under salt stress. Seeds of Nipponbare were incubated in distilled water (CK), 150 mM NaCl (Salt), and 150 mM NaCl + 0.1 mM SA (Salt + SA) for 1, 3, and 5 days. The amount of Na^+^ (**A**) and K^+^ (**B**) accumulated in seeds from the different treatments and incubation times are compared, along with the Na^+^/K^+^ ratio (**C**). Data are presented as means ± SE. Different lowercase letters indicate significant differences between treatments (*p* < 0.05).

Under salt stress, Na^+^ accumulation increased sharply, while K^+^ accumulation decreased significantly, increasing the Na^+^/K^+^ ratio (Figure 3). The accumulation of Na^+^ decreased by 9.85% and 6.64% at days 1 and 3 of treatment with 0.1 mM SA, respectively. Although SA had no significant effect on K^+^ accumulation, it effectively reduced Na^+^/K^+^ by 11.29% and 11.20%, alleviating ion stress from rice seeds (Figure 3). However, as compared to salt stress, SA treatment had no significant effect on the accumulation of Na^+^ and K^+^ in seeds at day 5 of incubation, and thus exhibited no significant difference in the Na^+^/K^+^ ratio either.

### 2.3. Exogenous SA Promoted the Expression of OsHKTs under Salt Stress

**Figure 4:**
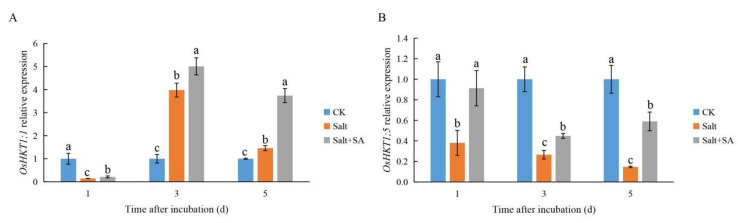
Effects of exogenous salicylic acid (SA) on the expression of *OsHKT1;1* and *OsHKT1;5* in rice seed under salt stress. Seeds of Nipponbare were incubated in distilled water (CK), 150 mM NaCl (Salt), and 150 mM NaCl + 0.1 mM SA (Salt + SA) for 1, 3, and 5 days. The expression of *OsHKT1;1* (**A**) and *OsHKT1;5* (**B**) in seeds subjected to the different treatments and incubation times are compared. Data are presented as means ± SE. Different lowercase letters indicate significant differences between treatments (*p* < 0.05).

In order to explore the mechanism of exogenous SA maintaining a Na^+^/K^+^ balance in rice seeds under salt stress, we detected the expression of *OsHKT1;1* and *OsHKT1;5* after 1 to 5 days of different treatments, and the results are shown in Figure 4. Compared with salt stress, the expression levels of *OsHKT1;1* and *OsHKT1;5* were increased by 46.46%, 25.90%, and 156.40%; and 139.81%, 68.73%, and 301.18%, respectively, after 1 to 5 days of Salt + SA treatment.

### 2.4. Exogenous SA Increased the Activity of Antioxidant Enzymes and Promoted the Scavenging of ROS under Salt Stress

**Figure 5:**
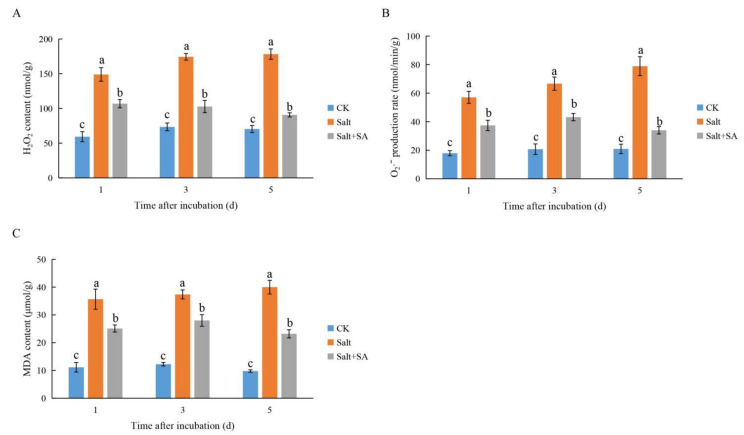
Effects of exogenous salicylic acid (SA) on the accumulation of H_2_O_2_, O_2_^−^, and malonaldehyde (MDA) in rice seeds under salt stress. Seeds of Nipponbare were incubated in distilled water (CK), 150 mM NaCl (Salt), and 150 mM NaCl + 0.1 mM SA (Salt + SA) for 1, 3, and 5 days. The accumulation of H_2_O_2_ (**A**), O_2_^−^ (**B**), and MDA (**C**) in seeds subjected to the different treatments and incubation times are compared. Data are presented as means ± SE. Different lowercase letters indicate significant differences between treatments (*p* < 0.05).

**Figure 6:**
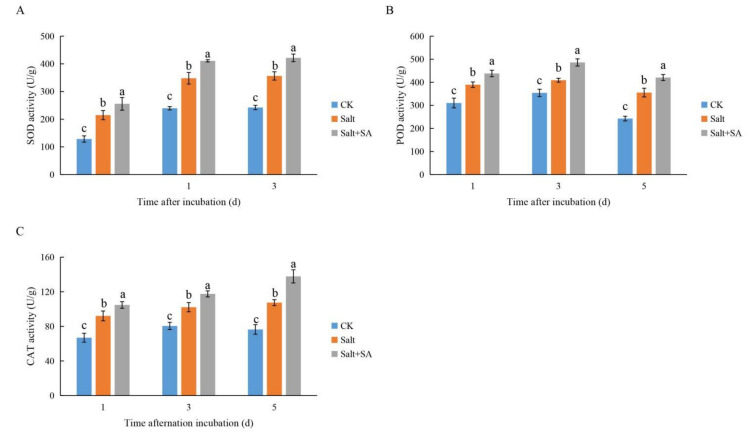
Effects of exogenous salicylic acid (SA) on superoxide dismutase (SOD), peroxidase (POD), and catalase (CAT) activities in rice seeds under salt stress. Seeds of Nipponbare were incubated in distilled water (CK), 150 mM NaCl (Salt), and 150 mM NaCl + 0.1 mM SA (Salt + SA) for 1, 3, and 5 days. The activity of SOD (**A**), POD (**B**), and CAT (**C**) in seeds subjected to the different treatments and incubation times are compared. Data are presented as means ± SE. Different lowercase letters indicate significant differences between treatments (*p* < 0.05).

As shown in Figure 5 and Figure 6, 150 mM NaCl significantly increased the accumulation of ROS and malondialdehyde (MDA) in rice seeds during 1 to 5 days of treatment followed by induction of antioxidant enzyme activity. Exogenous SA further improved the activity of antioxidant enzymes and promoted the elimination of ROS; this effect was the most significant after 5 days of incubation. As compared to CK, the content of hydrogen peroxide (H_2_O_2_), superoxide anion (O_2_^−^), and MDA improved by 153.23%, 101.76%, and 308.62%, respectively. The activities of SOD, POD, and CAT improved by 47.13%, 46.47%, and 40.66%, respectively, under salt stress. Upon treatment with 0.1 mM SA, the SOD, POD, and CAT activities increased by 18.31%, 18.36%, and 28.24%, respectively. Furthermore, ROS were eliminated, and the content of H_2_O_2_, O_2_^−^, and MDA decreased by 49.08%, 56.89%, and 41.97%, respectively.

### 2.5. Exogenous SA Positively Regulated GAs and ABA Homeostasis under Salt Stress

**Figure 7:**
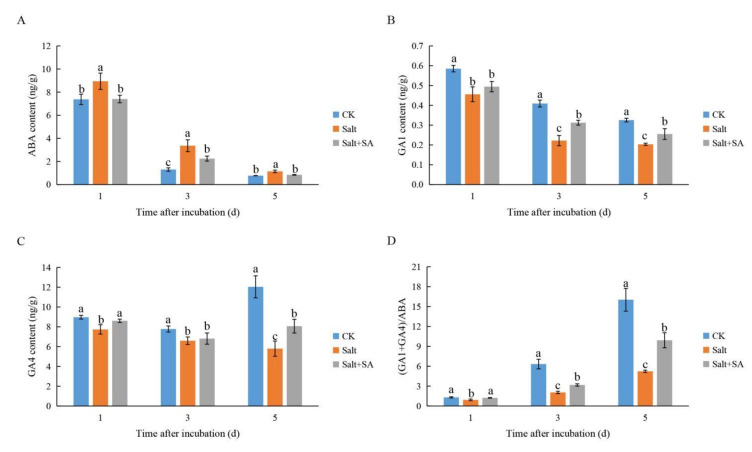
Effect of exogenous salicylic acid (SA) on endogenous hormones in rice seeds under salt stress. Seeds of Nipponbare were incubated in distilled water (CK), 150 mM NaCl (Salt), and 150 mM NaCl + 0.1 mM SA (Salt + SA) for 1, 3, and 5 days. The content of abscisic acid (ABA) (**A**), GA1 (**B**), and GA4 (**C**) in seeds from the different treatments and incubation times are compared, along with values of (GA1 + GA4)/ABA (**D**). Data are presented as means ± SE. Different lowercase letters indicate significant differences between treatments (*p* < 0.05).

Determination of endogenous hormone content showed that salinity increased ABA content, decreased GA1 and GA4 contents, and decreased the (GA1 + GA4)/ABA ratio in rice seeds. On the contrary, treatment with 0.1 mM SA decreased ABA content, increased GA1 and GA4 content, and increased the ratio of (GA1 + GA4)/ABA (Figure 7). After incubation for 1, 3, and 5 days, ABA content in seeds treated with 150 mM NaCl increased by 21.29%, 157.73%, and 48.10% as compared to the control; GA1 content decreased by 22.11%, 45.75%, and 37.54%, while GA4 content decreased by 13.80%, 15.18%, and 51.86%, respectively. Treatment with exogenous SA for 1, 3, and 5 days reduced ABA content by 17.18%, 33.14%, and 26.47%; increased GA1 content by 8.47%, 40.81%, and 25.42%; and increased GA4 content increased by 11.30, 3.25, and 39.11%, respectively.

### 2.6. Exogenous SA Regulated GA and ABA Metabolism under Salt Stress

**Figure 8:**
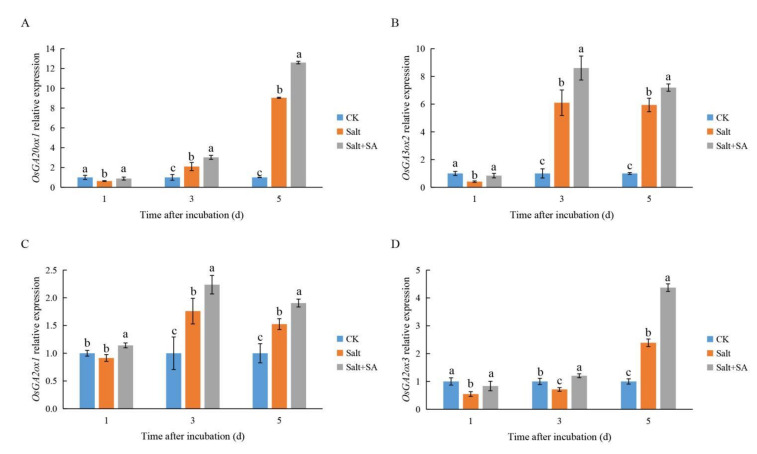
Effects of exogenous salicylic acid (SA) on gibberellic acid (GA) biosynthesis and inactivation of associated gene expressions under salt stress. Seeds of Nipponbare were incubated in distilled water (CK), 150 mM NaCl (Salt), and 150 mM NaCl + 0.1 mM SA (Salt + SA) for 1, 3, and 5 days. The expressions of GA biosynthetic genes (**A**) *OsGA20ox1* and (**B**) *OsGA3ox2* and inactivation genes (**C**) *OsGA2ox1* and (**D**) *OsGA2ox3* were compared. Data are presented as means ± SE. Different lowercase letters indicate significant differences between treatments (*p* < 0.05).

**Figure 9:**
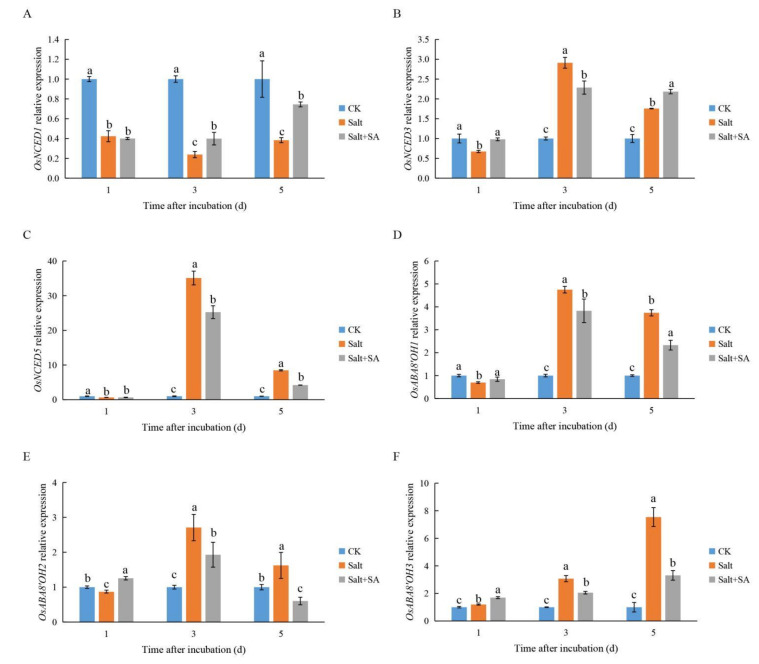
Effects of exogenous salicylic acid (SA) on abscisic acid (ABA) biosynthesis and expression of ABA catalyzing genes under salt stress. Seeds of Nipponbare were incubated in distilled water (CK), 150 mM NaCl (Salt), and 150 mM NaCl + 0.1 mM SA (Salt + SA) for 1, 3, and 5 days. The expressions of ABA biosynthetic genes (**A**) *OsNCED1*, (**B**) *OsNCED3*, and (**C**) *OsNCED5* and catalytic genes (**D**) *OsABA8′OH1*, (**E**) *OsABA8′OH2*, and (**F**) *OsABA8′OH3* were recorded. Data are presented as means ± SE. Different lowercase letters indicate significant differences between treatments (*p* < 0.05).

To explore the mechanism by which SA affected the content of GA and ABA during seed germination, the expression of GA and ABA biosynthesis and catabolism genes was monitored after different incubation periods (Figure 8 and Figure 9). Gene expression results showed that after incubation for a day, salinity significantly inhibited the expression of the GA biosynthesis genes *OsGA20ox1* and *OsGA3ox2*, while exogenous SA enhanced the expression of these two genes. Salinity induced the expression of *OsGA20ox1* and *OsGA3ox2* from day 3 onwards, while SA treatment further enhanced their expression (Figure 8A,B). Similarly, the GA inactivation gene *OsGA2ox1* was induced by salinity after incubation for 3 days, while *OsGA2ox3* was induced after incubation for 5 days. Exogenous SA treatment also promoted the expression of *OsGA2ox1* and *OsGA2ox3* (Figure 8C,D).

The expression levels of ABA biosynthesis and catabolism genes under different treatments showed that salinity and SA influenced the expression of ABA metabolism in rice seeds. The expression of *OsNCED1* was inhibited by salinity, while exogenous SA alleviated this inhibitory effect (Figure 9A). The expression levels of *OsNCED3* and *OsNCED5* increased from day 3 onwards, especially the expression of *OsNCED5*, which was enhanced by 35.10×; exogenous SA effectively inhibited the expression of these two ABA biosynthesis genes (Figure 9B,C). However, the expression levels of the ABA catabolism genes *OsABA8’OH1*, *OsABA8’OH2*, and *OsABA8’OH3* were significantly enhanced under salt stress, while exogenous SA inhibited their expression (Figure 9D–F).

### 2.7. Exogenous SA Alleviated the Inhibition of α-Amylase Activity by Salinity and Increased the Accumulation of Soluble Sugars

**Figure 10:**
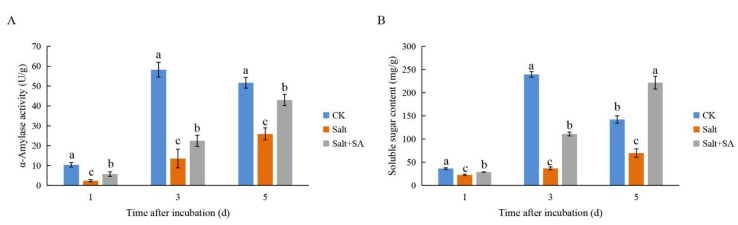
Effects of exogenous SA on α-amylase activity (**A**) and soluble sugar content (**B**) under salt stress. Seeds of Nipponbare were incubated in distilled water (CK), 150 mM NaCl (Salt), and 150 mM NaCl + 0.1 mM SA (Salt + SA) for 1, 3, and 5 days. Data are presented as means ± SE. Different lowercase letters indicate significant differences between treatments (*p* < 0.05).

Seed germination depends on the hydrolysis of starch by α-amylase to provide energy. Treatment with 150 mM NaCl for 1, 3, and 5 days inhibited α-amylase activity, and decreased the starch hydrolysis rate and the content of soluble sugar in rice seeds (Figure 9). The activity of α-amylase was significantly increased by 143.35%, 66.42%, and 66.31% at days 1, 3, and 5 after 0.1 mM SA treatment (Figure 10A), respectively, which promoted the hydrolysis of starch and increased soluble sugar content by 27.45%, 203.31%, and 218.04%, respectively (Figure 10B).

## 3. Discussion

Salinity is an important factor that may limit crop productivity by inhibiting seed germination [59]. As an important plant growth regulator, exogenous SA has often been reported to be involved in plant responses to salt stress [60,61]. In this study, treatment with 150 mM NaCl significantly inhibited the germination of rice seeds, while exogenous application of SA significantly increased the GR, GP, and GI of rice under salt stress (Figure 1). Jini and Joseph [58] also asserted that treatment with exogenous SA improved rice seed germination under conditions of salinity. Interestingly, rice cultivars with different sensitivities to salinity respond differently to SA under salt stress [62]. By comparing the germination characteristics of Nipponbare (salt-sensitive) and Huaidao 5 (salt-tolerant) varieties, it was observed that SA promoted the germination of the salt-sensitive rice genotype more significantly under salt stress, while it had no stimulatory effect on the salt-tolerant genotype. Furthermore, the germination levels of Nipponbare under salt stress were comparable to those of Huaidao 5 in the application of exogenous SA (Figure 1). In line with this, Lee et al. [63] found that exogenous SA had a two-sided effect on Arabidopsis seed germination under salt stress, whereby it promoted germination at concentrations of <10 μm, while it inhibited germination at concentrations >100 μm under salt stress. In this study, a low concentration (0.1 mM) of SA promoted germination, while a higher concentration (1 mM) of SA had no significant effect on the germination of rice seeds under salt stress (Figure 1).

Salinity induces Na^+^ accumulation and K^+^ efflux, destroys ion homeostasis, causes ion toxicity, and inhibits plant growth and development [64,65]. Chen et al. [66] reported that salinity increased Na^+^ accumulation and decreased K^+^ accumulation in cotton seeds, which severely inhibited seed germination. In this study, treatment with 150 mM NaCl significantly increased the accumulation of Na^+^ during the different stages of rice germination, while reducing the content of K^+^ and the Na^+^/K^+^ ratio, which was similar to the results of Jini and Joseph [58]. *OsHKT1;1* and *OsHKT1;5* play important roles in Na^+^ efflux, and their increased expression contributes to maintaining ion balance [67,68]. In this study, exogenous SA reduced the accumulation of Na^+^ in rice seeds by activating the expression of *OsHKT1;1* and *OsHKT1;5*, and maintained the balance of Na^+^/K^+^ under salt stress. However, contrary to the findings of Jini and Joseph [58], treatment with SA reduced the accumulation of Na^+^, but had no significant effect on the content of K^+^ (Figure 3). This discrepancy in findings may be due to the fact that their experiments were conducted with seedlings rather than seeds. The accumulation of Na+ in seeds decreased significantly after treatment with SA at days 1 and 3, but there was no significant difference between the SA + NaCl and NaCl treatments on day 5. We speculated that the radicle length was longer, and thus, the absorption area was higher after 5 days of SA treatment, which led to increased accumulation of Na^+^.

ROS accumulation is commonly observed during seed imbibition [47]. Luo et al. [45] demonstrated that salinity-induced ROS accumulation inhibited seed germination. In this study, treatment with 150 mM NaCl induced an accumulation of H_2_O_2_ and O_2_^−^, aggravated lipid membrane peroxidation, and increased MDA accumulation (Figure 4), which inhibited seed germination. Plants maintain redox homeostasis under salt stress by scavenging excess ROS through their antioxidant systems [69]. Previous studies on potato and wheat have shown that SA treatment enhances the activities of SOD, POD, and CAT, and may alleviate oxidative damage under salt stress [70,71]. In this study, treatment with 0.1 mM SA reduced the accumulation of H_2_O_2_, O_2_^−^, and MDA by enhancing the activities of SOD, POD, and CAT (Figure 5 and Figure 6) and maintaining ROS homeostasis in seeds, which led to improved germination of rice seeds under conditions of salinity.

Decreasing bioactive GA content is an important pathway to inhibition of seed germination under salt stress [72]. Previously, there was no consensus on the effects of salinity on the expression of genes related to GA metabolism in rice seeds. Liu et al. [24] found that the expression levels of all GA biosynthetic and inactivation genes were higher under salt stress than in the control. The authors suggested that salinity induced the expression of GA inactivation genes and decreased the content of bioactive GAs; upregulation of the GA biosynthesis gene may be a result of the negative feedback due to the salinity-induced lack of bioactive GA. However, Li et al. [73] found that the expression of GA biosynthesis genes in salt-sensitive rice was inhibited by salinity, while the expression of GA inactivation genes was induced by salinity after 6–48 h of incubation. After incubation for 72 h, the expression trend was reversed, suggesting that salinity decreased the content of bioactive GAs in seeds. On the contrary, we found that exogenous SA could restore the content of GA1 and GA4 in rice seeds under salt stress to a certain extent (Figure 7B,C). Liu et al. [24] also showed that the application of exogenous SA increased GA content in *Limonium bicolor* seeds. Furthermore, monitoring the expression of GA metabolic genes, we found that the expression of two key genes in GA biosynthesis, *OsGA20ox1* and *OsGA3ox2*, was inhibited by salinity within a day of incubation; however, expression was induced under conditions of salinity from day 3 onwards. They also implied that upregulated GA biosynthetic gene expression may be a consequence of negative-feedback regulation of NaCl-induced bioactive GA deficiency [24]. The expression of these two genes in the Salt + SA treatment was higher than that in the Salt treatment at all points in time (Figure 8A,B), suggesting that SA may increase the content of bioactive GA in rice seeds by enhancing the expression of GA biosynthetic genes. Liu et al. [74] also suggested that SA may enhance the expression of *GA20ox* and *GA3ox* in *Limonium bicolor* seeds under salt stress. Although exogenous SA activates the expression of the GA biosynthesis genes *OsGA20ox1* and *OsGA3ox2*, it also enhances the expression of the GA inactivation genes *OsGA2ox1* and *OsGA2ox3* (Figure 8). Measurement of hormone content indicated GA1 and GA4 to be significantly higher in rice seeds under the Salt + SA treatment as compared to salt stress alone (Figure 7B,C). In conclusion, the key pathway by which exogenous SA improves bioactive GA content in rice seeds is by enhancing the expression of GA biosynthetic genes, rather than inhibiting the expression of genes that deactivate GA production.

ABA is also an important plant hormone that affects seed germination. Salinity can inhibit seed germination by positively regulating ABA synthesis [72,75]. However, the effect of salinity on ABA metabolism in rice seeds has not been reported. Previous studies have shown that the expression of the ABA biosynthetic gene *OsNCED5* is upregulated under salt stress, which promotes the accumulation of ABA [76]. In this study, we found that the expression of *OsNCED5* changed more than that of *OsNCED1* and *OsNCED3* under salt stress (Figure 9A–C), indicating that *OsNCED5* may be a key gene related to salinity in rice seed germination. Treatment with 0.1 mM SA decreased the expression of *OsNCED5*, indicating that SA reduces ABA content in rice seeds by inhibiting ABA biosynthesis (Figure 7A and Figure 9C). Similarly, in *Limonium bicolor*, SA was reported to reduce ABA content and promote seed germination by inhibiting the expression of ABA biosynthetic genes [74]. Kong et al. [77] demonstrated that exogenous H_2_O_2_ reduced ABA content in cotton seeds under salt stress by inhibiting the expression of ABA biosynthesis genes and enhancing the expression of ABA catabolism genes. However, in this study, the expression of the ABA catabolism genes *OsABA8’OH1*, *OsABA8’OH2*, and *OsABA8’OH3* was enhanced by salinity, while their expression decreased upon treatment with exogenous SA (Figure 9D–F). This was attributed to the SA treatment lessening the accumulation of ABA, which led to a weaker induction of ABA catabolism genes as compared to under salt stress.

α-Amylase is an important hydrolase associated with seed germination; it promotes the hydrolysis of starch to soluble sugars and provides energy for seed germination [78]. Previous studies have shown that salt stress inhibits starch hydrolysis by reducing α-amylase activity during seed germination, thereby delaying seed germination [24,73]. This study also proved that as compared to the control, seeds treated with 150 mM NaCl had significantly reduced α-amylase activity and soluble sugar content. Furthermore, treatment with 0.1 mM SA effectively alleviated the inhibition of α-amylase activity and promoted the hydrolysis of starch to soluble sugars (Figure 10). This may have been due to the increased content of bioactive GAs in the rice seeds as a result of the application of exogenous SA, which, in turn, activated the transcription of α-amylase [24].

## 4. Materials and Methods

### 4.1. Plant Materials and Germination Treatments

Seeds of the Japonica rice varieties Nipponbare and Huaidao 5 (*Oryza sativa* L.) were used for this study. Nipponbare is sensitive to salt, while Huaidao 5 is salt-tolerant. Both types of rice seeds were provided by the College of Plant Protection at Shandong Agricultural University, Shandong Province, China. Rice seeds were disinfected with 75% ethanol and 2.5% (*v*/*v*) NaClO according to the method of Wang et al. [79]. The disinfected rice seeds were germinated in 90 mm Petri dishes on two layers of filter paper immersed in 10 mL distilled water (CK); 150 mM NaCl (≈8.766‰; NaCl); or 150 mM NaCl with 0.1, 0.5, or 1 mM SA (NaCl + 0.1SA, NaCl + 0.5 SA, and NaCl + 1 SA). Each treatment had five replicates and 30 seeds per replicate. Finally, the petri dishes were sealed with parafilm. All seeds were germinated for 1, 2, 3, 4, and 5 days in an artificial climate incubator under dark conditions at 28 °C.

### 4.2. Seed Germination Analysis

The number of seeds germinated in the different treatments at days 1, 2, 3, 4, and 5 was counted, and photos were taken after 5 days. GR was calculated at day 5. GP and GI were calculated using the following equations [80] with minor changes in GP calculation:

$$\mathrm{GR}\;\left(\%\right)\;=\;\frac{\mathrm{Total}\;\mathrm{number}\;\mathrm{of}\;\mathrm{germinated}\;\mathrm{seeds}\;}{\mathrm{Total}\;\mathrm{number}\;\mathrm{of}\;\mathrm{tested}\;\mathrm{seeds}\;}\;\times \;100$$

$$\mathrm{GP}\;\left(\%\right)\;=\;\frac{\mathrm{Number}\;\mathrm{of}\;\mathrm{germinated}\;\mathrm{seeds}\;\mathrm{within}\;\mathrm{four}\;\mathrm{days}}{\mathrm{Total}\;\mathrm{number}\;\mathrm{of}\;\mathrm{tested}\;\mathrm{seeds}\;}\times 100$$

$$\mathrm{GI}\;=\sum \left(\frac{\mathrm{Gt}}{\mathrm{Dt}}\right)$$

where Gt denotes the number of seeds on day t, and Dt means day t.

### 4.3. Determination of Na^+^ and K^+^ Content

Nipponbare seeds were germinated in distilled water, 150 mM NaCl, or 150 mM NaCl + 0.1 mM SA. After 5 days of incubation, Na^+^ and K^+^ content was determined in seeds by flame photometry as described by Wang et al. [81].

### 4.4. Quantification of ROS Levels and Antioxidant Enzyme Assays

Nipponbare seeds were germinated in distilled water, 150 mM NaCl, or 150 mM NaCl + 0.1 mM SA. After 5 days of incubation, 100 mg of germinated seeds was used to determine H_2_O_2_, O_2_^−^, and malondialdehyde (MDA) content. The activities of SOD, POD, and CAT were estimated using commercial kits from Solarbio Science & Technology Co., Ltd. (Beijing, China).

### 4.5. RNA Isolation, cDNA Synthesis, and Gene Expression Analysis

Imbibition seeds were collected from the different treatments at days 1, 3, and 5 and rapidly frozen in liquid nitrogen. Total RNA was extracted from the seeds using an RNeasy Plant Total RNA Isolation Kit (Qiagen, Valencia, CA, USA) according to the manufacturer’s instructions. The first strand of cDNA was synthesized from 1 μg of total RNA in a 20 μL reaction volume using Rescript II RT SuperMix reverse transcriptase (Nobelab, Beijing, China). Quantitative real-time PCR was performed in 96-well blocks on a CFX96 Touch Real-Time PCR System (Bio-Rad, Hercules, CA, USA) using the 2× SYBR Premix UrTaq II (Nobelab, Beijing, China) with a total reaction volume of 20 μL. Each reaction was performed as independent biological triplicates. Primer sequences used for qRT-PCR are presented in Table 1.

### 4.6. Quantification of GA1, GA4, and ABA in Rice Seeds

Nipponbare seeds were germinated in distilled water, 150 mM NaCl, or 150 mM NaCl + 0.1 mM SA. After 5 days of incubation, 100 mg of germinated seeds were collected for determination of ABA, GA1, and GA4 contents by an LC-MS system. Endogenous ABA was determined using the methods of Liu et al. [82], and quantification of endogenous GAs was performed as described by Chen et al. [83].

### 4.7. Quantitative Assay for α-Amylase Activity

Nipponbare seeds were germinated in distilled water, 150 mM NaCl, or 150 mM NaCl + 0.1 mM SA. After 5 days of incubation, 100 mg of germinated seeds were collected for determination of α-amylase activity as described by Wang et al. [84]. The process was performed in triplicates.

### 4.8. Determination of Soluble Sugar Content in Rice Seeds

Nipponbare seeds were germinated in distilled water, 150 mM NaCl, or 150 mM NaCl + 0.1 mM SA. After 5 days of incubation, 100 mg of germinated seeds were collected for determination of soluble sugar content by a soluble sugar content assay kit (Solarbio, Beijing, China) according to the manufacturer’s protocol.

### 4.9. Statistical Analysis

Statistical analysis was carried out with SPSS 22.0 software by using one-way analysis of variance (ANOVA), followed by Tukey’s test. Differences were considered significant at *p* ≤ 0.05. Data are presented here as means ± SD from at least three measurements unless indicated otherwise.

## 5. Conclusions

In this study, 150 mM NaCl significantly inhibited the germination of salt-sensitive rice (Nipponbare), while a dilute solution of SA (0.1 mM) helped maintain the ion balance, ROS content, and endogenous hormone homeostasis in rice seeds. It also alleviated the inhibitory effect of salinity on α-amylase activity and promoted hydrolysis of starch to soluble sugars, which collectively promoted the germination of rice seeds under salt stress. The findings of this study are of great significance to ensure the improved yield of crops in saline soils.

## Figures and Tables

**Table 1:** Primers used for quantitative real-time PCR in this study.

Gene	Accession Numbers	Forward Primer (5′→3′)	Reverse Primer (5′→3′)
*OsActin*	LOC4333919	GACTCTGGTGATGGTGTCAGC	GGCTGGAAGAGGACCTCAGG
*OsGA20ox1*	LOC4334841	CCACTACTTCCGGCGATTCTTCCAGCG	GACGTGGTCCTGGTGGAGGATGGTG
*OsGA3ox2*	LOC4337968	GGAGAGCAAGGCCGTGTATCAGG	CTCTCCTTGTCCTCTTCCTTCGCTAC
*OsGA2ox1*	LOC4337874	TGACGATGATGACAGCGACAA	CCATAGGCATCGTCTGCAATT
*OsGA2ox3*	LOC4325145	TGGTGGCCAACAGCCTAAAG	TGGTGCAATCCTCTGTGCTAAC
*OsNCED1*	LOC4330451	CTGGAGCACATGGAGCTAGTGCACTCC	CCGACGCCGAAGTAGCCGTACCTG
*OsNCED3*	LOC4333566	CCCCTCCCAAACCATCCAAACCGA	TGTGAGCATATCCTGGCGTCGTGA
*OsNCED5*	LOC9270250	TCATTCCAAAACACCTTCCA	TCCGGGGACCTCCTATGTAT
*OsABA8′OH1*	LOC9269675	AAGTACAGGTGGTCCACGTCCAA	CCAGCTTAGCTGATGCTAGTATTC
*OsABA8′OH2*	LOC4345810	GACGAGGTGGAGTACAGCCCGTTC	GGACACATCAGCCACCATCAGCAGTAG
*OsABA8′OH3*	LOC4347261	CAGTGTGGAAACAGATGGTTGC	CGGACTTCCCTTGAGGAAATAGA
*OsHKT1;1*	LOC9266695	TTCACCACTCTTGCGGCTATG	TGTTTGTAGCCAGTCTCCCCAG
*OsHKT1;5*	LOC4327757	CCACCTTTTCCTTTTCCATGC	GGTCTTCATCGGCAGAGCTTT

## Data Availability

Not applicable.
